# Proteomic evaluation and comparison of native and decellularized human saphenous vein extracellular matrices

**DOI:** 10.3389/fbioe.2026.1773372

**Published:** 2026-03-30

**Authors:** Essi M. Niemi, Tuula A. Nyman, Håvard Attramadal, Henrik Hoel, Antonio Rosales, Jonny Hisdal, Hanne Scholz

**Affiliations:** 1 Institute for Surgical Research, Rikshospitalet, Oslo University Hospital, Oslo, Norway; 2 Department of Vascular Surgery, Ullevål Hospital, Oslo University Hospital, Oslo, Norway; 3 Hybrid Technology Hub, Centre of Excellence, Institute of Basic Medical Sciences, University of Oslo, Oslo, Norway; 4 Institute of Clinical Medicine, Faculty of Medicine, University of Oslo, Oslo, Norway; 5 Department of Immunology, University of Oslo and Oslo University Hospital, Oslo, Norway; 6 Department of Transplantation Medicine, Oslo University Hospital, Oslo, Norway

**Keywords:** cardiovascular diseases, extracellular matrix, human saphenous vein, regenerative medicine, tissue decellularization, vascular basement membrane, vascular graft, vascular tissue engineering

## Abstract

**Background:**

The growing prevalence of cardiovascular diseases (CVDs) has created an increasing demand for alternative biologically functional vascular grafts. Among the explored tissue engineering strategies, decellularization provides a means to generate suitable acellular scaffolds from donor tissues with preserved complex extracellular matrix (ECM) architecture and vascular basement membrane (BM), both of which are critical for graft endothelialization and function. To assess decellularization efficiency and quality, proteomic profiling offers high-resolution characterization of tissue composition, enabling systematic analysis of extracellular matrix subgroups and residual proteins with the aid of open-access annotation tools, thus providing critical insights beyond conventional methods.

**Methods:**

Human saphenous vein segments were decellularized using a CHAPS-based protocol, and decellularization efficiency was evaluated by DNA and glycosaminoglycan (GAG) quantification, together with histological evaluation. A subset of samples (n = 9) was further subjected to label-free quantitative proteomic profiling using LC-MS/MS to assess ECM composition, BM integrity, and residual cellular content.

**Results:**

Decellularization led to 99.96% DNA removal and 53.78% GAG retention, with well-preserved tissue morphology. Decellularization efficiency was further assessed by label-free quantitative proteomics, which showed well-preserved ECM composition. Collagens, proteoglycans, and BM proteins were largely well-maintained, whereas ECM glycoproteins and matrisome-affiliated proteins were moderately reduced. The strongest decrease in protein abundance was observed among cellular proteins, and Gene Ontology analysis confirmed the reduction of key cellular protein groups.

**Discussion:**

In this study, we provide a comprehensive proteomic evaluation of decellularized human saphenous veins, demonstrating selective preservation and loss across ECM subgroups and cellular proteins. The findings highlight proteomics as a valuable complement to conventional assays for assessing decellularization efficiency and establishing a reference framework for optimizing protocols in vascular tissue engineering.

## 1 Introduction

According to the World Health Organization (2021), cardiovascular diseases (CVDs) are the leading cause of death worldwide, with the number of individuals affected by CVDs drastically increasing each year (Roth et al., 2020). CVDs cause life-threatening structural damage to vessels, such as narrowing and blockage, and small-diameter (<6 mm) vascular grafting is often required to replace or bypass these damaged vessel segments. Autologous vessel transplant is the gold standard, but it is not always available, making it a limited graft option; meanwhile, allograft vessels often lead to immune rejection, and synthetic grafts lack biocompatibility and compliance (Pashneh-Tala et al., 2016; Li et al., 2023).

Tissue decellularization has shown great potential for the development of bioengineered vascular grafts for bypass surgeries. Multiple inventive and efficient decellularization protocols using various methods and sourced from human (Teebken et al., 2009; Wilshaw et al., 2012; Kajbafzadeh et al., 2019; Mallis et al., 2020), bovine (Daugs et al., 2017; Lopera Higuita et al., 2022), equine (Böer et al., 2015), ovine (Zhao et al., 2010; Mogaldea et al., 2017), and porcine (Ma et al., 2017; Pu et al., 2018; Simsa et al., 2018) vascular tissues have been presented as potential grafting materials. However, despite significant efforts, human and especially animal-derived decellularized grafts are struggling to progress to clinical trials due to challenges of immunogenicity, donor-to-donor variability, and poor re-endothelialization potential for long-term patency (Kasravi et al., 2023).

The aim of decellularization is to remove DNA and cellular components to reduce the risk of cytotoxicity and immune rejection of the donated tissues while utilizing gentle techniques to preserve the integrity and architecture of the extracellular matrix (ECM) (Crapo et al., 2011; Gilpin and Yang, 2017). In vascular tissues, the preservation of the vascular basement membrane (BM), a specialized ECM layer underlying the endothelial cells in the vessel lumen, is crucial as it performs a key function in vascular cell attachment, migration, and tissue regeneration (Lopera Higuita et al., 2022). By retaining the vascular-specific biochemical and structural cues, these promote vascular cell repopulation and enhance the regenerative processes *in vivo* (Saldin et al., 2017; Henry et al., 2020; Durán-Rey et al., 2021).

To ensure the clinical and research applicability of decellularized tissues, it is essential to implement robust methods for defining and reporting the matrix quality after treatment. Traditional approaches, such as residual DNA quantification, biochemical assays, mechanical testing, and advanced microscopy, are effective and suitable methods for the initial evaluation of decellularization efficiency (Crapo et al., 2011). However, these lack the resolution needed to fully characterize the complexity of the tissue composition. For instance, studies have shown that low DNA content does not automatically correlate with the removal of cellular components (Li et al., 2016). As a result, proteomic profiling has gained significant interest as a tool for evaluating decellularized tissues as it provides a platform for high-resolution, comprehensive analysis of both the retained ECM components and the residual cellular proteins (Berger et al., 2020; Asthana et al., 2021). To further facilitate systematic data interpretation, open-access tools such as MatrisomeDB, Gene Ontology (GO) Knowledgebase, and PANTHER can be utilized to support protein classification and functional annotation, thus providing insights into the molecular composition and enabling the evaluation of compositional changes introduced by decellularization (Ashburner et al., 2000; Naba et al., 2016; Thomas et al., 2022).

The human saphenous vein, commonly harvested for bypass surgeries, serves as an ideal model for investigating venous vascular protein composition and the effects of decellularization, due to its accessibility, structural robustness, and clinical relevance. The aim of this study is to report the first in-depth proteomic profiling of healthy human saphenous veins to characterize and define the venous proteome, along with the ECM and BM compositions before and after decellularization.

In this study, we utilized proteomic analysis to characterize the effects of decellularization on the protein composition of human saphenous veins. Collagen and proteoglycans were largely well-preserved, while more soluble ECM proteins were markedly reduced, indicating the partial loss of non-fibrillar matrix components during decellularization. Residual cellular proteins, especially cytoskeletal proteins, were also detected, while other cellular proteins were considerably reduced, indicating variable resistance to removal. Beyond assessing the effectiveness of the decellularization process, this study expands on existing knowledge of the vascular protein profile to provide detailed insights into the compositional complexity of proteins critical for vascular tissue engineering.

## 2 Methods

### 2.1 Vein material

Human saphenous vein segments of varying lengths (1 cm–8 cm) were harvested from 20 male and one female patient, aged 46–80 years, who underwent elective coronary artery bypass graft surgery at Oslo University Hospital (Supplementary Table S1). The study was carried out with the approval of the Regional Committee for Medical and Health Research Ethics of Southeast Norway D (165664) and after obtaining written informed consent for the use in research. The vein segments were stored refrigerated at +4 °C in University of Wisconsin (UW) solution right after the surgery for maximum of 48 h. Upon receipt at the laboratory, the veins were washed once in saline to remove residual blood. Excess saline was removed using surgical non-woven cloth, and the veins were snap-frozen in liquid nitrogen at −80 °C until further processing. The storage time at −80 °C was a maximum of 3 months from the date of operation until processing. Due to the varied sizes of the segments, it was not possible to utilize all the surgical samples in all the analyses.

### 2.2 Decellularization

The vein samples were rapidly thawed to room temperature (RT) in phosphate-buffered saline (DPBS; Lonza Group AG, Basel, Switzerland), and half of each vein segment was designated for decellularization, while the other half was retained as the native control. The vein samples were then washed and prepared by longitudinally opening the veins and further sectioning them into approximately 1 cm^2^ pieces. The vein samples were decellularized in parallel in a series of solutions in Nalgene™ Straight-Sided Wide-Mouth Polycarbonate Jars (500 mL; Thermo Fisher Scientific, Oslo, Norway) with continuous shaking in a New Brunswick Scientific™ Innova™ 42 Incubator Shaker (New Brunswick Scientific Co., Inc., Edison, NJ, United States) and a buffer volume of 200 mL if not otherwise stated. First, the tissue pieces were washed in DPBS overnight at +4 °C with gentle agitation at 80 rpm to remove residual blood, followed by a wash in 100 mM ethylenediaminetetraacetic acid (EDTA; Sigma-Aldrich, Oslo, Norway) in DPBS, adjusted to pH 7.8, for 22 h in +37 °C with agitation at 110 rpm. Second, the pieces underwent a detergent wash in 8 mM 3-[(3-cholamidopropyl) dimethylammonio]-1-propanesulfonate (CHAPS, Abcam, Cambridge, United Kingdom) buffer supplemented with 1 M NaCl and 25 mM EDTA in DPBS for 22 h at +37 °C with agitation at 110 rpm, adjusted to pH 7.6, and the buffer was changed to fresh buffer after the first 3 h of incubation. The samples were washed overnight in DPBS at +4 °C with agitation at 80 rpm to efficiently remove the detergent residues from the tissue pieces.

Third, the vein pieces were incubated 2 × 3 h with 10 U/mL Benzonase® Nuclease (Sigma-Aldrich) supplemented with 1 mM MgCl_2_ in 50 mM Tris buffer (Sigma-Aldrich) at pH 8.1, +37 °C, with agitation at 100 rpm in smaller 100-mL volume sample jars with a tissue-to-buffer ratio of approximately 10 mg wet weight to 1 mL of Benzonase buffer. In the fourth and final step, immediately after the Benzonase washes, the tissue pieces were transferred to 2 M NaCl (Sigma-Aldrich) in DPBS hypertonic buffer to remove any remaining residual DNA, and the samples were incubated overnight at +4 °C with agitation at 80 rpm. To continue the washing process, the decellularized samples were washed for 2 days in DPBS supplemented with 1 x Gibco™ Antibiotic–Anti-mycotic (Thermo Fisher Scientific) at +4 °C with agitation at 80 rpm, with the DPBS replaced daily to remove any residual decellularization reagents. The samples were kept in gentle agitation throughout the protocol at low speed to facilitate movement, ensuring fresh contact between the tissues and reagents.

### 2.3 DNA quantification

To quantify the amount of remaining double-stranded DNA from the tissue, approximately 16 mg–20 mg of wet tissue was cut from the native and decellularized vein samples, and 1–3 replicates were taken from different sites of the tissue pieces, depending on the size of the received surgical segments. The tissue pieces were initially minced into smaller fragments using surgical scissors to facilitate more efficient solubilization and then dried using a Thermo Scientific Savant SpeedVac Concentrator SPD131DDA (Thermo Fisher Scientific) at 70 °C for 2 h, with the dry weights of the samples recorded. The samples were incubated at +4C sequentially in 15 % and 30 % sucrose solutions. The samples were then incubated at 56 °C until the tissue pieces were completely lysed for a maximum time of 3 h and vortexed every 30 min to enhance the lysis. After complete lysis, the samples were further processed and purified according to the manufacturer’s instructions. The purified DNA samples were quantified using a Quant-iT PicoGreen dsDNA Assay Kit (Thermo Fisher Scientific), according to the manufacturer’s instructions. The samples were excited at 485 nm, and the fluorescence emission intensity was measured at 520 nm using a POLARStar Omega Plate Reader (BMG Labtech, Ortenberg, Germany); all samples were measured in technical duplicates.

### 2.4 Glycosaminoglycan quantification

To quantify the preservation of sulfated glycosaminoglycans (GAGs), approximately 20 mg–25 mg of wet tissue was cut from the decellularized and native vein samples, and 2–3 replicates were taken from different sites of the tissue depending on the size of the pieces. The samples were dried using a SpeedVac concentrator at 50 °C for 3 h, and the dry weights of the samples were noted down. GAG quantification was performed using the Blyscan sGAG Assay (BioColor, Belfast, United Kingdom), with the tissues first rehydrated with 0.2 M sodium phosphate buffer at pH 6.4, according to the assay manual. The samples were digested in a papain extraction buffer (Sigma-Aldrich) at 65 °C for 3 h, as described in the manual, and further processed for the assay according to the manufacturer’s instructions. Absorbance of the samples was measured in technical duplicates at 650 nm using the Victor™ X5 Plate Reader (PerkinElmer, Waltham, MA, United States).

### 2.5 Histology staining

Samples were taken for cryosectioning and histology evaluation upon arrival from native tissue and after decellularization. First, the tissues were fixed for 48 h with 10% neutral buffered formalin (Chemi-Teknik AS, Oslo, Norway) and washed with PBS. The samples were incubated overnight at 4 °C in 15% and 30% sucrose solutions (Sigma-Aldrich) in PBS, then embedded in Tissue-Tek OCT compound (Sakura Finetek Norway AS, Oslo, Norway), snap-frozen in liquid nitrogen, and stored at −20 °C. Cross-sections measuring 8 μm were serially sectioned using CryoStar NX70 (Thermo Fisher Scientific), and the sections were collected in Fisherbrand™ Superfrost™ Plus Microscope Slides (Fisher Scientific, Oslo, Norway). The samples were further dyed using the Vector Laboratories Hematoxylin and Eosin Staining Kit (BioNordika, Oslo, Norway), Alcian Blue 8GX pH at 2.5 with Nuclear Fast Red Solution, and Masson’s Trichrome Staining Kit, followed by mounting with Eukitt® Quick-hardening Mounting Medium (all from Sigma-Aldrich).

### 2.6 Sample preparation for proteomics

For proteomic profiling, 10 samples were prepared from native tissues and their decellularized counterparts in duplicates with a three-step lysis protocol. Frozen samples were first pulverized in a mortar with a pestle in liquid nitrogen, followed by 40-min incubation with 200 μL radioimmunoprecipitation assay (RIPA) lysis buffer supplemented with 1 × HALT protease and phosphatase inhibitor cocktail (Thermo Fischer Scientific) in ice with gentle mixing with a pipette, and then centrifuged at 8,000 *g* for 10 min. The supernatants were pipetted to new 1.5-mL Eppendorf tubes and kept on ice. The remaining insoluble fractions of the samples were lysed again in 200 μL of a buffer containing 7 M urea and 2 M thiourea, supplemented with 5% CHAPS, 1 mM EDTA, and 1 × HALT in 30 mM Tris-HCl buffer at pH 8.5, for 40 min at RT with gentle mixing during incubation, and then centrifuged. The supernatant from this second lysis step was combined with the RIPA lysate, gently mixed, and kept on ice. Total protein content in the samples was measured using the Bio-Rad Protein Assay (Bio-Rad Laboratories Inc., Hercules, CA, United States), according to the manufacturer’s Microtiter Plate Protocol, and absorbance was measured at 595 nm using Victor™ X5.

An amount of 25 µg of protein from all lysates was precipitated with 70% acetonitrile onto magnetic beads (MagReSyn Amine, Resyn Biosciences). The proteins were washed on the beads with 100% acetonitrile and 70% ethanol and then resuspended in 50 µL of 50 mM ammonium bicarbonate containing 10 mM DTT for the reduction of cysteines. The samples were incubated at 37 °C for 60 min. Then, 50 µL of 30 mM IAA in 50 mM ammonium bicarbonate was added to alkylate proteins, and the samples were incubated at RT in the dark for 30 min. An amount of 1 µg of trypsin was added to each sample for overnight on-bead protein digestion at 37 °C. The peptides thus obtained were desalted on C18 stage tips (Affinisep) before LC-MS/MS analysis.

### 2.7 Proteomic analysis

The desalted peptide samples (500 ng/sample) were analyzed using a nanoElute UHPLC system coupled to a timsTOF fleX mass spectrometer (Bruker Daltonics, Bremen, Germany) via a CaptiveSpray ion source. Peptides were separated on a 25 cm reversed-phase C18 column (1.6 µm bead size, 120 Å pore size, and 75 µm inner diameter; Ion Optics) with a flow rate of 0.3 μL/min and a solvent gradient from 0% to 35% B in 60 min. Solvent B was 100% acetonitrile in 0.1% formic acid, and solvent A was 0.1% formic acid in water. The mass spectrometer was operated in the data-dependent parallel accumulation–serial fragmentation (PASEF) mode. Mass spectra for MS and MS/MS scans were recorded between 100 and 1,700 m/z. Ion mobility resolution was set to 0.85 Vs/cm–1.30 Vs/cm over a ramp time of 100 m. Data-dependent acquisition was performed using 10 PASEF MS/MS scans per cycle with a near 100% duty cycle. A polygon filter was applied in the m/z and ion mobility space to exclude low m/z singly-charged ions from PASEF precursor selection. An active exclusion time of 0.4 min was applied to precursors that reached 20,000 intensity units. Collisional energy was ramped stepwise as a function of ion mobility.

MS raw files were submitted to MaxQuant software (version 2.0.1.0) (Cox and Mann, 2008) for protein identification and quantification. Parameters were set as follows: carbamidomethylation as a fixed modification and protein N-acetylation and methionine oxidation as variable modifications. The first search error window was 20 ppm, and the main search error window was 4.5 ppm. Trypsin without the proline restriction enzyme option was used, with two allowed miscleavages. Minimal unique peptides were set to 1, and the FDR allowed was 0.01 (1%) for peptide and protein identification. The ‘match between runs’ (MBR) option was enabled. The UniProt human database was used (downloaded from UniProt Sept. 2020). Generation of reversed sequences was selected to assign FDR rates. LC-MS/MS files from technical duplicates were merged in MaxQuant search. The mass spectrometry proteomics data have been deposited to the ProteomeXchange Consortium *via* the PRIDE partner repository (Perez-Riverol et al., 2025) under the dataset identifier PXD068043.

Additional proteomics data analysis and filtering were carried out using Perseus software (version 1.6.15.0). Sample N6 had a markedly lower number of protein identifications and total intensity in the MaxQuant search than the other samples; thus, the sample pair N6/D6 was considered an outlier and not included in the quantitative analysis (Supplementary Figure S1). In Perseus, MaxQuant entries ‘REVERSE’ and ‘CONTAMINANT’ were removed, protein intensities were log_10_-transformed, and data were filtered to require that a protein be identified in at least 5/9 replicates within a group (native or decellularized). Missing values were imputed using a constant of 0.

### 2.8 Data processing and statistics

For the DNA and GAG quantification, 1–3 biological replicates were analyzed. Data were presented as the mean ± SD and calculated using GraphPad Prism (v9.3.1). Statistical differences were determined by Wilcoxon signed-rank test for the comparison of the groups, and the significance level was set to a *p*-value <0.05. ECM components from the proteome were identified and classified using the MatrisomeDB annotator tool (Naba et al., 2016), which comprises both *in silico* and *in vivo* data on the tissue matrisome. The complete protein ID list was processed through the SynGO ID conversion tool (Koopmans et al., 2019), a compatible gene list for GO annotations. GO Knowledgebase (Ashburner et al., 2000; Alexander et al., 2023) and the PANTHER Tool (Thomas et al., 2022) were utilized to annotate the native and decellularized proteomes with 18 selected GO terms. For the sample-level analysis of CC GO term basement membrane (GO:0005604), the native and decellularized samples (n = 9 for both groups) were z-score-normalized for each protein separately within the two groups and visualized as a clustered heatmap for comparison using GraphPad Prism. For the glycosaminoglycan-binding protein analysis, log_2_ fold change values were calculated as log_2_-transformed ratios of mean protein abundances between decellularized and native tissues (n = 9 per group).

## 3 Results

### 3.1 Evaluation of decellularization efficiency

**FIGURE 1:**
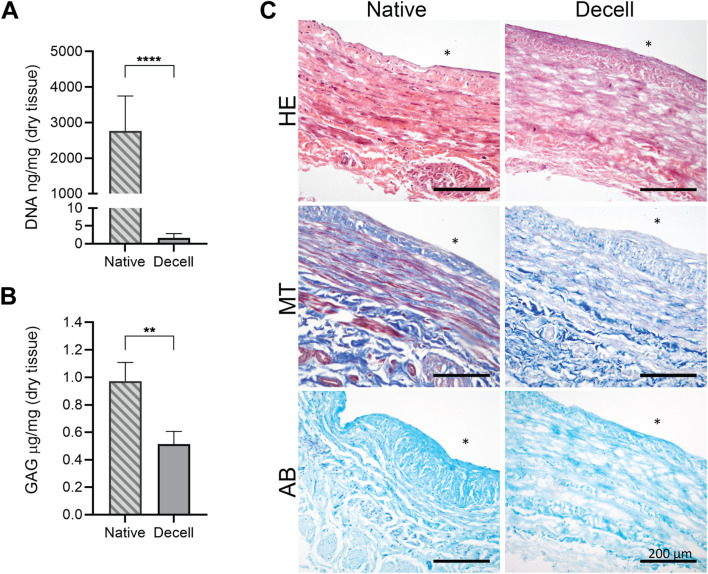
Evaluation of the decellularized vein tissue compared to the native tissue. **(A)** The DNA amount was measured using the PicoGreen assay (n = 16), which revealed an average of 99.96% DNA removal from the samples. **(B)** Remaining GAG quantity in the tissue was determined using the Blyscan assay (n = 9), which revealed that an average of 53.78% GAG quantity was restored after decellularization. **(C)** Histology staining for HE, MT, and AB confirmed the removal of nucleus. Data are presented as the mean ± SD; the native vein sample group is named Native; the decellularized group is named Decell. Statistical differences between the native and decellularized samples were analyzed using the Wilcoxon signed-rank test, ****p < 0.0001; **p = 0.0039 (* indicates the luminal side of the vein tissue; scale bar: 200 µm).

The vein segments were decellularized with a CHAPS-based decellularization protocol combined with a Benzonase nuclease wash step. The decellularization showed almost complete removal of DNA from the samples. DNA content was measured from the dry weight of the decellularized vein segments (n = 16) and compared with their native counterparts using the PicoGreen assay, which showed an average DNA removal of 99.96% (Figure 1A). The residual amount of DNA in the decellularized sample group was 1.60 ± 1.24 ng/mg (mean ± SD) compared with the native sample group, which had 2,767.58 ± 979.63 ng/mg DNA.

The remaining GAG quantity in the tissue was determined using the Blyscan assay from the dry weight of the decellularized vein segments (n = 9) and their native counterparts. The analysis revealed 53.78% retention of GAG contents after decellularization (Figure 1B). The amount of GAG in the decellularized sample group was 0.51 ± 0.09 μg/mg compared with 0.97 ± 0.14 μg/mg in the native sample group.

To qualitatively assess decellularization efficacy and cell removal, along with the preservation of the vascular tissue structure, histological evaluation was performed using hematoxylin and eosin (HE), Masson’s Trichrome (MT), and Alcian Blue (AB) staining (Figure 1C). The histological analysis confirmed the removal of nuclei with all the staining methods and showed well-retained tissue structure. AB staining confirmed GAG preservation in the tissue, whereas MT staining of decellularized tissue showed reduced binding of Biebrich Scarlet-Acid Fuchsin solution, which typically stains acidophilic elements such as cytoplasm, muscle, and collagen. After treatment with phosphomolybdic acid, the dye diffuses from collagen, leaving only the muscle fibers stained red. These muscle fibers were visible in native tissue but absent in decellularized samples, providing clear evidence of the removal of vascular smooth muscle cells from the tunica media layer of the vascular wall.

### 3.2 Overall proteomic composition of human saphenous vein

We performed high-resolution label-free quantitative proteome analysis for 20 samples, 10 native samples and their 10 decellularized counterparts. All samples were prepared and analyzed by LC-MS/MS in duplicates. Data from the duplicates were merged during data analysis. Evaluation of proteomics data quality revealed that sample N6 had a markedly lower number of protein identifications and clustered separately from the other native samples in principal component analysis (PCA) (Supplementary Figure S1); thus, the sample pair N6/D6 was excluded from further analysis. For quantitative comparison of the native and decellularized vein proteomes, the proteomics data were filtered to include only proteins reproducibly identified in at least five out of nine samples within a group, native or decellularized. In addition, to enable the identification of low-abundant proteins from the samples, we used the match between run (MBR) function in the MaxQuant database search.

**FIGURE 2:**
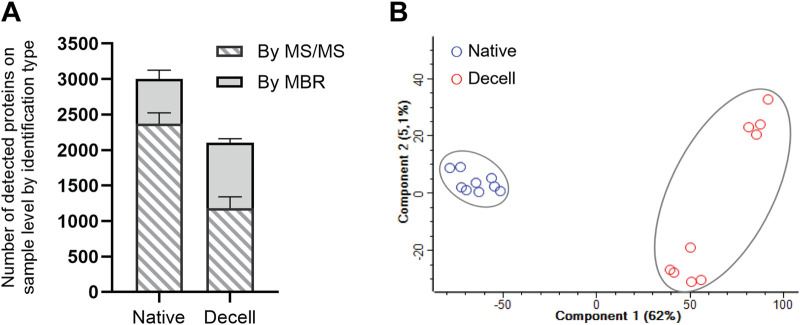
Number of identified proteins in the MS proteomic evaluation of native and decellularized sample groups after filtering based on the detection type and principal component analysis (PCA) of the native and decellularized vein proteomes. **(A)** The bar chart shows the average number of detected proteins based on the identification type, either by MS/MS or by MBR, within the two studied vein groups, native (Native, n = 9) and decellularized (Decell, n = 9). **(B)** The PCA plot show distinct clustering of the native and decellularized groups, indicating a distinct change in the composition after decellularization. Component 1 and component 2 explain 62% and 5.1% of the variance, respectively. Each point represents an individual sample, with distinct colors (blue, native; red, decellularized.

With this approach, we reproducibly identified a total of 3,089 proteins, of which 1,494 proteins were common across all the samples in both groups. A total of 3,083 and 2,293 unique proteins were reproducibly identified from the native and decellularized samples, respectively (Figure 2A; Supplementary Table S2A). At the individual sample level, an average of 2,373 proteins were detected by MS/MS in the 9 native samples, while 630 proteins were detected solely by the MBR feature (Supplementary Table S2B). In contrast, in the 9 decellularized samples, on average 1179 proteins were detected by MS/MS, while 925 proteins were detected only by matching (Supplementary Table S2C). In addition, the results show the removal of 898 proteins on average per sample after decellularization when the native samples and their decellularized counterparts were compared (native vein nr. 1 vs. decellularized vein nr. 1). When comparing the total intensities of samples in these two groups, the decellularized samples had an average total intensity of only 58.8% of their native counterparts, emphasizing the effect of the decellularization in removing proteins.

PCA (Figure 2B) indicated a clear separation between the native and decellularized sample groups, primarily explained by component 1, which accounted for 62.0% of the total variability. This separation emphasized the distinct effects of the decellularization protocol on the tissue before and after decellularization. The native samples showed a higher degree of homogeneity than the decellularized samples, which showed some internal heterogeneity within the group. Component 2, which accounted for 5.1% of the variability, captured this subtle heterogeneity introduced during the decellularization process.

To identify the most abundant proteins in the two groups, native and decellularized, the protein intensities for each detected protein were averaged across the nine samples within each group. The 200 most abundant detected proteins of the native and decellularized vein tissue groups are listed in Supplementary Tables S3A,B. Among the 200 most abundant proteins in both experimental groups, 148 were common between the two groups. For the 200 most abundant proteins in the native group, their combined intensity comprised 84.2% of the total intensity, while the remaining 15.8% corresponded to 2,883 different proteins. In contrast, the combined intensity of the top 200 proteins within the decellularized group covered 94.1% of the total intensity. The remaining 5.9% of the total intensity was comprised of 2,093 different proteins within the decellularized group. The increased proportion of the 200 most abundant proteins after decellularization indicates the removal of proteins, resulting in a proteome that is more compositionally concentrated. However, the remaining 5.9% comprises a wide range of potentially lower abundance proteins indicating still a high degree of complexity after decellularization.

### 3.3 Proteomic annotation of ECM, BM, and cellular proteins after decellularization

ECM is a fundamental structural and functional constituent of the venous wall, and different ECM proteins have distinct functions in the vessel wall layers. To understand the effects of decellularization on the venous ECM, the ECM components of the proteome were identified and classified with the MatrisomeDB annotator developed by Naba et al. (2016). The annotator classifies ECM proteins into two main categories, namely, the core matrisome, comprising collagens, proteoglycans, and ECM glycoproteins, and matrisome-associated proteins, which include ECM-affiliated proteins, ECM regulators, and secreted factors. The complete list of matrisome proteins identified by the MatrisomeDB annotator tool is provided in Supplementary Tables S4A, S4B for the native and decellularized sample groups, respectively.

**FIGURE 3:**
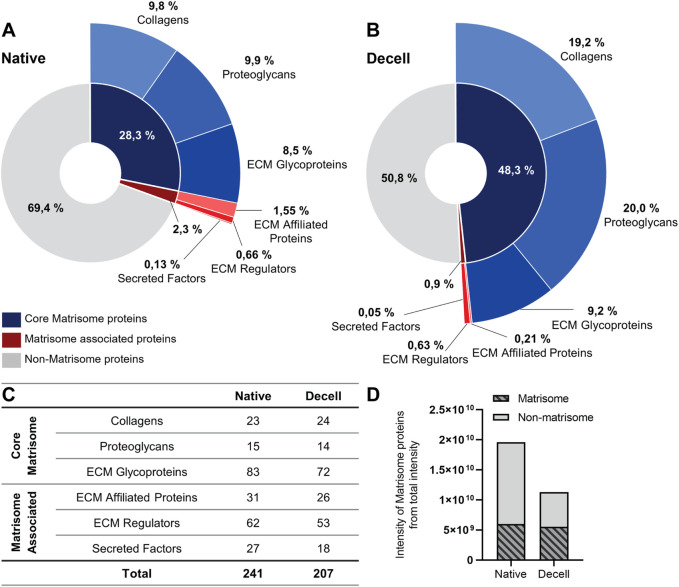
ECM composition of native and decellularized tissue annotated using the MatrisomeDB tool. A total of 241 matrisome proteins were identified from the native samples, and 207 matrisome proteins were identified from the decellularized samples. The charts **(A,B)** represent the intensity fraction of the ECM proteins from the total protein intensity of the **(A)** native and **(B)** decellularized groups. Blue color represents the core matrisome proteins and its three subcategories, namely, collagens, proteoglycans, and ECM glycoproteins, and red color represents the matrisome-affiliated proteins and its three subcategories, namely, ECM-associated proteins, ECM regulators, and secreted factors. **(C)** Table presenting the number of identified proteins within the matrisome protein categories for both groups. **(D)** Bar chart representing the intensity of the matrisome proteins from the total intensity of the proteins within the native and decellularized groups.

The MatrisomeDB annotator tool identified 241 ECM proteins in the native sample group, of which 121 were classified as core matrisome proteins and 120 as matrisome-associated proteins. The abundance of the core matrisome proteins was 28.3% and that of the matrisome-associated proteins was 2.4% of the total intensity of the native tissue proteome (Figure 3A). The core matrisome proteins consisted of 23 collagens, 15 proteoglycans, and 83 ECM glycoproteins and the matrisome-associated proteins consisted of 31 ECM-affiliated proteins, 62 ECM regulators, and 27 secreted factors (Figure 3C).

A total of 207 matrisome proteins were identified from the decellularized sample group, of which 110 were core matrisome proteins and 97 were matrisome-associated proteins (Figure 3B). The share of the core matrisome proteins was 48.3%, and that of the matrisome-associated proteins was 0.9% of the total intensity of the decellularized proteome. Within the detected ECM proteins from the decellularized proteome, core matrisome proteins consisted of 24 collagens, 14 proteoglycans, and 72 ECM glycoproteins and the matrisome-associated proteins consisted of 26 ECM-affiliated proteins, 53 ECM regulators, and 18 secreted factors (Figure 3C). The number of identified matrisome proteins from the decellularized group was slightly lower than that of the native proteome, but ECM protein abundance was maintained at a similar level to that of the native group, indicating well-preserved ECM after decellularization (Figure 3D). When compared to the native tissue, the intensities of collagens and proteoglycans especially remained largely consistent after decellularization, indicating minimal loss and effective preservation of the major ECM constituents.

The results indicate minimal changes in collagen content after decellularization as the 10 most abundant collagen alpha chain types were the same in both groups, namely, COL6A3, COL6A1, COL6A2, COL14A1, COL18A1, COL4A1, COL1A2, COL4A2, COL1A1, and COL12A1. Among these, collagen VI (COL6A1, COL6A2, and COL6A3) exhibited a well-preserved profile, remaining the most abundant collagen type detected in both groups. Essential BM-related collagens and network-forming collagen IV (COL4A1 and COL4A2) showed well-preserved profiles, whereas multiplexin collagen XVIII (COL18A1) showed lower abundance following decellularization. FACIT collagens XII and XIV (COL12A1 and COL14A1), which regulate fibril assembly and interaction with other matrix proteins, showed an equally well-preserved profile. However, the most abundant ECM proteins in connective tissues, including collagen I (COL1A1 and COL1A2), fibrillar collagens III and V (COL3A1, COL5A1, and COL5A2), and glycoprotein elastin (ELN), were detected at consistently low levels in both native and decellularized samples. This likely reflects their inherent resistance to solubilization during sample preparation for proteomic analysis, leading to their underrepresentation in the dataset.

Similarly to collagens, the intensities of proteoglycans in the decellularized group remained comparable to those of the native group following decellularization. The seven most abundant proteoglycans were all highly relevant key proteins for vascular tissue, represented in both the native and decellularized groups. Six of these were small leucine-rich proteoglycans (SLRPs), namely, prolargin (PRELP), biglycan (BGN), lumican (LUM), decorin (DCN), asporin (ASPN), and mimecan (OGN), and one was specifically important basement membrane proteoglycan perlecan (HSPG2). Other preserved relevant proteoglycans for vascular tissues were fibromodulin (FMOD) and versican (VCAN). However, the ratios of the different proteoglycans varied between the two groups, indicating selective retention or depletion of specific proteoglycans during the decellularization process.

**FIGURE 4:**
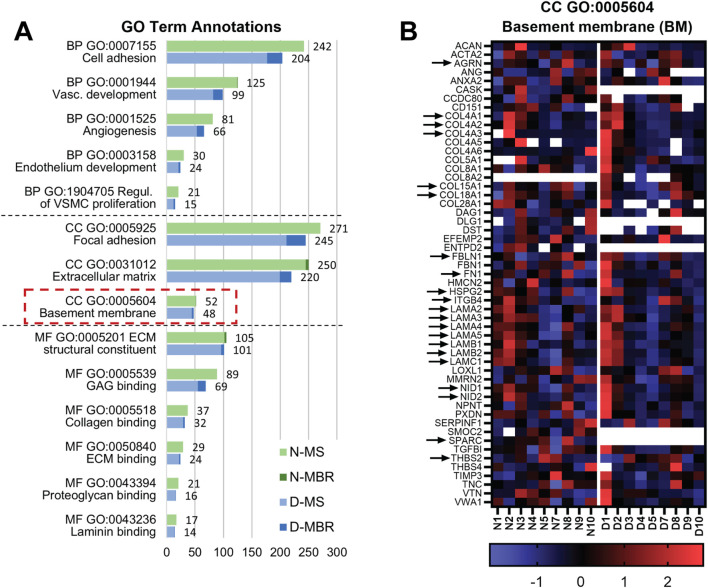
GO term annotations for evaluation of the preserved proteome relevant for vein tissue. **(A)** Graph illustrating the number of detected proteins in the native and decellularized samples based on GO term annotations. The selected GO terms for evaluation represent protein groups that play a relevant role in function and health of vascular tissue and its ECM based on biological process (BP), cellular component (CC), and molecular function (MF) annotations. The basement membrane (BM) GO:0005604 has been highlighted with the red dash-lined box and further analyzed. **(B)** Heatmap illustrating the preservation of BM proteins after decellularization. The native and decellularized samples (n = 9 for both groups) were z-score-normalized for each protein separately within the two groups and visualized as a clustered heatmap for the observation of patterns of preserved proteins in decellularized samples compared to those in their native counterparts. Red indicates that a protein was expressed in higher quantity within the group from the average, and blue indicates lower abundance within the group compared to that in other samples. Missing protein identifications have been indicated with white. Black arrows highlight the central proteins related to BM health.

Within the core matrisome proteins, ECM glycoproteins were more affected by the decellularization process than proteoglycans and collagens, exhibiting slightly reduced intensity. Among the 10 most abundant glycoproteins in both native and decellularized groups, eight were shared, namely, fibronectin 1 (FN1), tenascin-C (TNC), laminins (LAMA5, LAMB2, and LAMC1), transforming growth factor beta inducible (TGFBI), periostin (POSTN), and nidogen-1 (NID1). Most of these top-identified and well-retained glycoproteins were further identified in the GO term analysis as highly relevant proteins for healthy vascular BM function (Figure 4B). Other identified vital preserved ECM glycoproteins for cell–ECM interactions and ECM integrity from the decellularized group were agrin (AGRN), fibrillin-1 (FBN1), and fibulins (FBLN1, FBLN2, and FBLN5). Additionally, thrombospondins (THBS2 and THBS4) and von Willebrand factor (vWF) were equally identified after decellularization, which are proteins essential for regulating vascular cell function and blood coagulation. However, connective tissue growth factor (CTGF), an important controller of cell homeostasis and angiogenesis, was only identified in native samples.

Matrisome-associated proteins are low abundant, mostly soluble proteins within the tissue, that interact with the core matrisome proteins and are subcategorized into three groups depending on the purpose: ECM-affiiliated proteins, ECM regulators and screted factors. The results showed a diminished overall profile, particularly in terms of intensity, highlighting the importance of identifying matrisome-associated proteins that persist after decellularization and are critical for vascular function rather than prioritizing the most abundant proteins. The results revealed the preservation of central proteins involved in angiogenesis and tissue remodeling, such as fibroblast growth factor 2 (FGF2), matrix metalloproteinases (MMP2 and MMP4), angiopoietin-like 2 (ANGPTL2), and S100 proteins (S100A8 and S100A9). The function of these proteins is also closely related to many similarly retained pro- and anti-fibrinolytic proteins that are critical in wound healing, such as plasminogen (PLG), tissue plasminogen activator (PLAT), thrombin (F2), annexins (ANXA2 and ANXA5), antithrombin III (SERPINC1), and factors XII and XIII (F12 and F13A1). Additionally, many proteins important for ECM integrity and collagen–elastin remodeling were similarly retained, including procollagen-lysine, 2-oxoglutarate 5-dioxygenases (PLOD1 and PLOD3), lysyl oxidase (LOX), lysyl oxidase-like 1 (LOXL1), and tissue inhibitors of metalloproteinases (TIMP2 and TIMP3).

Next, the preservation of the different proteins closely related to vascular-specific functions after decellularization was annotated using the GO and PANTHER knowledge bases. The selected GO term annotations highlight protein groups with key roles in vascular tissue function and ECM health, although they are not limited only to ECM proteins. We compared the number of proteins identified in native *versus* decellularized tissues (Figure 4A), highlighting the retention of a significant number of proteins post-decellularization, particularly those with GO annotations closely related to the ECM. Additionally, the use of MBR in protein detection, as shown in Figure 4A revealed more comparable protein profiles between native and decellularized groups as some proteins may have decreased in abundance post-decellularization but remained at low levels across most samples. The removal or reduction of cellular proteins was more pronounced emphasized after decellularization in annotations related to cellular-associated functions, such as cell and focal adhesions and angiogenesis. Overall, the analysis indicated the complexity of the tissue was well-preserved after decellularization and showed the retention of protein groups essential for venous wall structure and function.

Among the selected GO terms, the focus was specifically on BM (GO:0005604) to analyze the preservation of proteins associated with vascular BM, an underlying structure of the vascular endothelium. Preservation of the BM is crucial for vascular health and endothelial cell function, adhesion, and migration. A total of 52 BM-related proteins were identified in the native group, while 48 were found in the decellularized group (Figure 4A). The decellularized samples exhibited the well-retained major structural network constituents of a healthy vascular BM (Figure 4B), such as collagen IV (COL4A1, COL4A2, and COL4A3), glycoprotein laminin isoforms 411 and 511 (LAMA4, LAMA5, LAMB1, and LAMC1), and their stabilizing nidogens (NID1 and NID2). Additionally, network-reinforcing proteins HSPG2 and AGRN were equally retained. Other essential proteins identified with roles in BM structural assembly and function and cell adhesion included collagens XV and XVIII (COL15A1 and COL18A1), FBLN1, laminin receptor integrin β4 (ITGB4), other laminins (LAMA2, LAMA3, and LAMB2), and THBS2. FN1 was also detected within this GO annotation, and although it is not a direct BM constituent, it contributes to BM assembly and endothelial adhesion and is, therefore, categorized as a BM-related protein. In contrast, osteonectin (SPARC), an important BM collagen-binding and pro-angiogenic factor, was depleted after decellularization.

In addition, to evaluate whether the observed reduction in sulfated GAG content following decellularization was associated with selective depletion of GAG-binding proteins, relevant proteins were compiled from MatrisomeDB-classified ECM proteins together with proteins annotated with the GO terms GAG binding (GO:0005539) and BM (GO:0005604). Log_2_ fold-change analysis comparing native and decellularized tissues (Supplementary Table S5) showed that the major structural ECM proteins were largely preserved or mildly enriched, especially proteoglycans including BGN, DCN, VCAN, and HSPG2, consistent with the MatrisomeDB analysis. In addition, key BM structural components such as type IV collagens and laminins were well-preserved. In contrast, several proteins associated with cellular or peri-cellular GAG-rich environments, including CD44, glypicans (GPC1, GPC4, and GPC6) and FGF2, were strongly reduced following decellularization, indicating that decellularization primarily removed cell-surface and peri-cellular GAG-binding proteins while preserving the structural ECM scaffold.

Proteomic analysis indicated that the abundance of matrisome proteins remained largely unchanged after decellularization, with the reduction in total protein content primarily affecting the non-matrisome proteins. To assess these changes, the native and decellularized non-matrisome proteomes were compared using selected GO terms related to key intracellular components, namely nucleus (GO:0005634), mitochondrion (GO:0005739), cytoskeleton (GO:0005856), and MHC protein complex (GO:0042611). Decellularization resulted in a marked reduction of non-matrisome protein content, with intensity decreasing to 42.1% of that in the native tissue (Figure 3D). In the native group, proteins were distributed as follows: 48.9% nucleus, 11.4% mitochondria, 32.8% cytoskeleton, and 0.17% MHC complex. In the decellularized group, the distribution was as follows: 38.2% nucleus, 11.4% mitochondria, 77.9% cytoskeleton, and 0.03% MHC complex, indicating a considerable reduction in most cellular groups, whereas the cytoskeletal protein abundance remained largely unchanged.

The most abundant non-matrisome proteins in both groups were primarily cytoplasmic proteins and key components of the contractile cytoskeletal microfilament network. In the native group, the 10 most abundant non-matrisome proteins were filamin-A (FLNA), actin filaments and myosin proteins (ACTA2, ACTG1, MYH11, and MYL6), desmin (DES), vimentin (VIM), calponin-1 (CNN1), hemoglobin subunit beta (HBB), and talin-1 (TLN1). Meanwhile, the 10 most intense non-matrisome proteins in the decellularized group included mostly actin filaments and myosin proteins (ACTA2, ACTG1, MYH11, MYH9, and MYH10), FLNA, DES, microtubules (TUBA1B and TUBB4B), and VIM.

## 4 Discussion

The limited availability of suitable autologous vessels for vascular grafting, combined with the poor performance and the high risk of graft failure of synthetic alternatives, has driven interest in assessing the suitability of decellularized arteries and veins from various sources as alternative bypass grafts. While decellularization effectively removes cellular components, it also changes the ultrastructure and protein composition of the treated tissue, potentially impacting graft performance (Böer et al., 2015; Daugs et al., 2017; Simsa et al., 2018). In this study, proteomic analysis of paired native and decellularized human saphenous veins provides a detailed assessment of ECM and BM protein retention and loss, offering molecular insight into how the process reshapes the composition of this highly clinically relevant conduit.

The most commonly used detergents, such as CHAPS, sodium dodecyl sulfate (SDS), Triton-X 100, and sodium deoxycholate (SDC), are widely reported to be efficient decellularization reagents when combined with endonucleases. However, no clear gold standard exists for vascular tissue decellularization as vascular tissues vary considerably in origin, thickness, and structure, and the published studies also report contrasting outcomes regarding decellularization efficiency and the resulting tissue quality (Daugs et al., 2017; Simsa et al., 2018; Mallis et al., 2020). Therefore, optimization is required to achieve a balance between effective cell removal and the preservation of ECM complexity. If the reagents are too harsh, they might cause undesirable effects, such as strong reduction of GAGs, denaturation of collagens, disruption of tissue ultrastructure and ECM, and even cytotoxicity (Crapo et al., 2011; Mendibil et al., 2020; Simsa et al., 2018).

In this study, veins were efficiently decellularized using a CHAPS- and Benzonase-based protocol adapted from published methods for thinner tissues, such as amniotic membrane, vascular tissue, and lung tissue (Tsuchiya et al., 2014; Daugs et al., 2017; Haupt et al., 2018; Henry et al., 2020; Mallis et al., 2020; Mendibil et al., 2020), and optimized with ovine jugular vein tissue (data not shown) to ensure efficient removal of DNA while preserving tissue complexity. CHAPS is a mild zwitterionic detergent that combines the properties of ionic and non-ionic detergents, and when optimized well, it has been shown to better preserve the matrix ultrastructure and key ECM components, including elastin, collagens, laminins, fibronectin, and GAGs, supporting improved biocompatibility in subsequent implantation studies compared to that with harsher detergents (Tsuchiya et al., 2014; Daugs et al., 2017; Simsa et al., 2018; Shin et al., 2019; Henry et al., 2020). Benzonase was selected as a suitable endonuclease as it has been shown to be highly efficient in removing DNA and, in addition, is available in a GMP-compliant form, supporting future translational applicability (Simsa et al., 2018; Vafaee et al., 2018).

The decellularization steps using CHAPS and Benzonase were designed to preserve ECM and tissue integrity while enabling efficient cell removal and minimizing unintended protease activity. In brief, EDTA washes were used to weaken cell–cell and cell–matrix adhesions and suppress metal-dependent proteases, followed by CHAPS treatment to solubilize cellular membranes and release intracellular contents, and Benzonase incubation was carried out to digest residual nucleic acids (Tsuchiya et al., 2014; Haupt et al., 2018; Vafaee et al., 2018). The protocol alternated between incubation at 4 °C and 37 °C, where lower temperatures reduced endogenous enzymatic activity and physiological temperatures enabled effective detergent and nuclease function. EDTA supplementation during detergent treatment further limited the metal-dependent protease activity, and reagent refreshing during CHAPS treatment promoted the removal of released cellular components while maintaining reagent effectiveness. Benzonase incubations were intentionally kept short to minimize prolonged exposure to 37 °C while still enabling efficient nucleic acid digestion. Nevertheless, some degree of protein degradation during detergent-based decellularization cannot be fully excluded and should, therefore, be considered when interpreting tissue composition and downstream proteomic results.

Mass spectrometry-based proteomics has emerged as a powerful tool for comprehensively characterizing protein composition and changes after decellularization (Giobbe et al., 2019; Berger et al., 2020; Asthana et al., 2021; Diedrich et al., 2024). In this study, native and decellularized human saphenous veins were subjected to high-resolution, label-free quantitative proteomics to evaluate the tissue quality and define the venous ECM proteome. This profiling revealed a considerable reduction in both the number and abundance of proteins after decellularization.

To enhance the in-depth analysis of the proteome, we utilized the MBR function in MaxQuant to enhance the sensitivity of our proteomic profiling and facilitate the detection of low-abundant proteins. The MBR feature improves data completeness by transferring peptide identifications across LC-MS/MS runs, thereby reducing missing values and enabling a more comprehensive protein profile for each sample (Yu et al., 2021). Similar use of MBR detections to assess the effects of decellularization on tissue proteomes has not been reported previously. In our study, the application of MBR substantially increased the detection of low-abundance proteins, especially ECM glycoproteins, matrisome-associated factors, cellular proteins, and GO term annotations. This enhanced sensitivity was particularly useful for analyzing decellularized samples, where direct MS/MS identifications decreased from 2,373 to 1,179 following decellularization, while MBR detections increased from 630 to 925, thereby preserving insights into the remaining proteome complexity despite a shift toward fewer, highly abundant proteins.

The MatrisomeDB annotator tool identified the loss of 11 core matrisome proteins and 23 matrisome-associated proteins after decellularization while still indicating the well-preserved abundance of ECM matrisome proteins. Together with GO term annotations, these findings strongly indicate that many proteins relevant to vascular ECM function and structure were retained, as all selected relevant categories showed only a slight decrease in protein numbers, indicating that no specific group was disproportionately affected by decellularization. This further indicates that the majority of removed proteins were non-matrisome-related proteins, with the total amount of non-matrisome proteins, mainly cellular proteins, reduced by 57.9%. These presented outcomes align with the study’s objective to remove cellular proteins while preserving the complexity of venous tissue ECM.

Despite the loss of some matrisome proteins, the analysis revealed well-preserved profiles of collagens and proteoglycans, closely resembling those of the native proteome in both composition and abundance. Proteoglycans, together with collagens, form the functional and physical ECM backbone of vascular tissue, which is highly crosslinked, rendering it resistant to the disruptive effects of decellularization. Among the identified proteins, the SLPRs are especially important for collagen matrix organization and significantly contribute to overall tissue function and stiffness (Matheson et al., 2005; Theocharis et al., 2016). In addition, PRELP and HSPG2 also perform crucial functions in anchoring the BM to the underlying connective tissue in conjunction with collagen VI and maintaining the endothelial layer integrity and function, making them highly essential to preserve for enhancing luminal health (Bengtsson et al., 2002; Kalluri, 2003; Groulx et al., 2011).

In contrast, consistently low detection levels of fibrillar collagens I, III, and V and elastin across all samples indicate that certain solubilization-resistant ECM proteins might be underrepresented in the results. These proteins, including large aggregating proteoglycans, are highly resistant to the effects of decellularization, but they are also resistant to protein solubilization, posing a known challenge in sample preparation for proteomic analysis of connective tissues (Dapic et al., 2019). Therefore, for sample preparation after pulverization, two complementary solubilization buffers were used as they exhibited distinct capabilities in solubilizing different proteins. Urea–thiourea solubilizes mainly larger cytoskeletal and extracellular regional proteins, while RIPA solubilizes mostly cytoplasmic, mitochondrial, and nuclear proteins (Ngoka, 2008; Peach et al., 2015). While this presented a three-step solubilization process that yielded the smallest insoluble fraction from the tested methods, it is possible that this introduces bias in the findings toward a higher proportion of cellular proteins, particularly in the decellularized group, as similarly reported by others (Xu and Shi, 2014; Barallobre-Barreiro et al., 2016; Calle et al., 2016; Naba et al., 2016).

In addition to the proteoglycan content analysis, it was essential to evaluate the effects of the decellularization on GAGs as each proteoglycan consists of a core protein covalently linked to one or more GAGs, together forming a functional unit. GAG content was quantified separately due to their significant contribution to ECM assembly, organization, and function, particularly within the BM (Uhl et al., 2020). The quantification revealed a reduction in GAG content following decellularization. However, GAGs and ECM glycoproteins are also present on cell surfaces, particularly within the endothelial glycocalyx layer, where they perform a critical function in cell signaling, permeability, and adhesion (Foote et al., 2022). The observed decrease partly reflects the removal of cellular and peri-cellular GAG-associated components, consistent with the log_2_ fold-change analysis, which showed a marked reduction of cell-associated GAG-binding proteins after decellularization. However, depletion of these proteins alone is unlikely to fully explain the reduced sulfated GAG content, indicating that the decellularization process may also partially extract or cleave GAG chains from otherwise preserved proteoglycan core proteins.

In addition, ECM glycoproteins were the most affected of the core matrisome proteins, although several functionally important members were still preserved and showed well-retained profiles. Glycoproteins possess a complex modular structure and serve numerous functions within the ECM and resident vascular cells, such as ECM assembly, remodeling, and the promotion of cell–ECM interactions (Hynes and Naba, 2011; Aamodt and Grainger, 2016). In connection, the GO term annotation for BM helped to identify the proteins critical for vascular tissue health and function, particularly those associated with maintaining the vascular endothelium. Most BM proteins, in addition to collagens and HSPG2, are glycoproteins such as laminins and nidogens. Proteins involved in BM organization, including FN1 and ITGB4, are especially relevant to maintain as they facilitate endothelial cell adhesion on the luminal surface (Kalluri, 2003; Yousif et al., 2013).

Matrisome-associated proteins showed a more reduced profile as many are soluble, sensitive to decellularization reagents, and challenging to prepare for mass spectrometry (Naba et al., 2016). Their preservation complements the core matrisome, particularly the BM complexity, as these perform a crucial function in supporting ECM functions, including promoting cell adhesion and migration. In vascular grafting applications, re-endothelization or recellularization of the graft lumen is critical, as the lumen is a blood-contacting surface. Healthy endothelium acts as an anti-thrombogenic barrier, preventing the exposure of thrombogenic ECM proteins that could trigger a clotting cascade, leading to thrombosis upon transplantation (Radke et al., 2018). Additionally, functional endothelium reduces the risk of inflammatory responses, calcification, and the formation of intimal hyperplasia. Complications such as these are frequent in biological and synthetic vascular grafts that fail to support proper endothelialization, thus highlighting the importance of well-preserved BM (Pashneh-Tala et al., 2016; Mallis et al., 2020).

In our previous study (Sämfors et al., 2022) we demonstrated the central role of laminin in enhancing endothelial cell adhesion and viability on biomaterial surfaces intended for vascularized tissues. In the study, natural mouse laminin was covalently conjugated to the internal hollow channel network of a nanocellulose–alginate hydrogel device, a biomaterial that has inherently low cell-adhesive properties. The channels were chemically modified using sodium periodate oxidation, followed by covalent bioconjugation of laminin to enhance endothelial cell adhesion. Laminin-functionalized channels supported robust human umbilical vein endothelial cell (HUVEC) adhesion by day 4 and sustained viable endothelial layers by day 14, whereas non-functionalized controls showed minimal cell adhesion.

Although the abundance of non-matrisome proteins was markedly reduced, the remaining substantial number of these proteins contrasts with the near-complete removal of DNA, emphasizing the need to evaluate the types of cellular proteins. The GO term annotations for the four cellular protein groups revealed a substantial reduction in the proportion of nuclear and mitochondrial proteins and a particularly reduced share of MHC complex proteins. This reduction in the MHC complex proteins can be considered as a positive indication of lowered immunogenicity, thereby potentially enhancing the material’s suitability for allogeneic transplantation (Manon et al., 2025).

The annotated cytoskeletal proteins accounted for 77.9% of the non-matrisome protein intensity, consistent with the 10 most abundant non-matrisome proteins identified after decellularization. These 10 proteins form a dynamic contractile network of intracellular filaments anchored to the transmembrane integrins and further connected to the underlying BM and neighboring cells (Dos Remedios et al., 2003; Janke and Magiera, 2020). This connection plays a crucial role in mechanotransduction, the process by which cells sense and respond to mechanical stimuli, thus enabling them to adapt to changing blood flow conditions and regulate vasoreactivity across the vessel wall (Wang et al., 2009; Butler, 2016). Given their integral role in the mechanotransduction and their strong linkage with extracellular structures, it is plausible that these connections contribute to their increased resistance to removal during the decellularization process.

The balance between sufficient cell removal and ECM preservation remains a challenge. It has been shown that although CHAPS is a milder detergent that preserves tissue complexity, its effects are quite surfactant and may lead to increased cellular residues compared to those with other detergents. Tsuchiya et al. (2014) compared CHAPS-based protocols with lung tissue in a rat subcutaneous implantation model. One of the protocols in the study used a near-neutral pH CHAPS wash, which preserved ECM and GAG content effectively but resulted in the retention of more cellular remnants. In contrast, the same protocol with higher pH removed cellular material more effectively but caused greater ECM damage and loss of GAGs (Tsuchiya et al., 2014). *In vivo* implantation of these tissues revealed that samples treated with the low-pH detergent wash induced less fibrosis and immune cell infiltration than the high-pH protocol, which could indicate that preserving tissue complexity may favor cell survival and promote desirable cellular behavior at implantation and during recellularization, potentially outweighing the adverse effects of the residual cellular debris, especially in the case of allografts (Böer et al., 2011; Tsuchiya et al., 2014; Kasravi et al., 2023). The controversy of low residual DNA but high number of cellular proteins calls for further studies in order to better understand the influence of these residues on the recellularization potential and subsequent use of the tissue, especially for grafting purposes (Calle et al., 2016; Li et al., 2016; Berger et al., 2020). Ultimately, the goal is to achieve a level of decellularization sufficient to avoid eliciting negative host responses while preserving the essential biological cues to support recellularization and constructive tissue remodeling, which are considered fundamental processes in tissue engineering for restoring native tissue function (Swinehart and Badylak, 2016).

## 5 Conclusion

Our study provides a comprehensive label-free quantitative proteomic analysis of clinically relevant decellularized human saphenous vein. We characterized the preservation and loss of key ECM and BM proteins that are critical for vascular health while also profiling the composition of key residual cellular proteins. The use of MaxQuant’s MBR feature increased the depth of our analysis, enabling the detection of low-abundance proteins and contributing to a more comprehensive characterization of the vascular proteome. These findings underscore the importance of systematic proteomic profiling for refining decellularization protocols to achieve optimal scaffold quality and recellularization potential. Ultimately, the knowledge generated here provides a reference framework to advance the safety and translational potential of decellularized venous grafts for clinical applications.

## Data Availability

The mass spectrometry proteomics data have been deposited to the ProteomeXchange Consortium via the PRIDE partner repository with the dataset identifier PXD068043.
