# Supplementary material for: Proteomic evaluation and comparison of native and decellularized human saphenous vein extracellular matrices
Source: Front Bioeng Biotechnol. 2026 Mar 30;14:1773372. doi: 10.3389/fbioe.2026.1773372 (PMC13071391; doi:10.3389/fbioe.2026.1773372)
Supplement: Supplementary file 6 [file Image1.pdf]

Supplementary Figure 1.

A)

Sample	Identifications per sample	Total intensity
N1	2936	1,55E+10
N2	2990	1,97E+10
N3	3060	2,41E+10
N4	3005	1,81E+10
N5	2966	1,82E+10
N6	2450	1,06E+10
N7	2950	1,65E+10
N8	3052	2,29E+10
N9	3073	1,94E+10
N10	3068	2,21E+10
D1	2348	1,57E+10
D2	2326	1,29E+10
D3	2365	1,19E+10
D4	2074	8,54E+09
D5	2020	7,43E+09
D6	2397	1,23E+10
D7	2434	1,16E+10
D8	2456	1,49E+10
D9	1951	9,89E+09
D10	1996	8,35E+09

B) Imputation with constant=0 | Standard imputation in Perseus

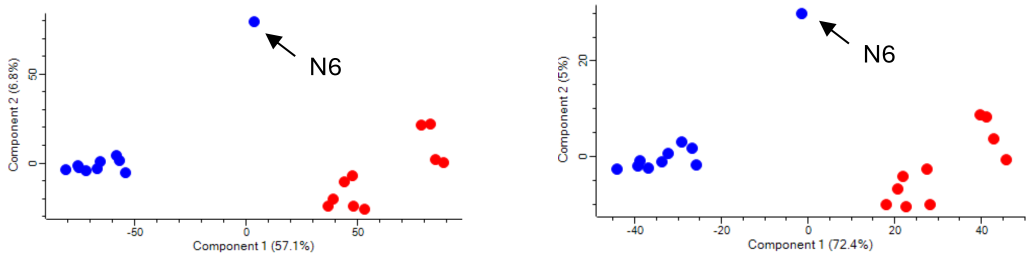

C)

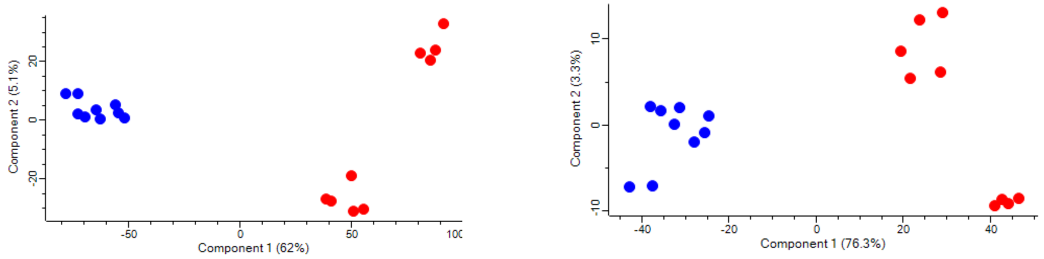

Blue = Native
Red = Decellularized

Supplementary Figure 1. Proteomics data quality evaluation

A) Overview of protein identification results

B) Principal component analysis of the full proteome dataset using two different imputation methods to replace missing values; left panel shows PCA using imputation with constant=0 and right panel shows PCA using imputation with random values from low-end of normal distribution using default settings in Perseus

C) PCA plots after removal of sample pair N6/D6
